# Associations between menopausal hormone therapy and sleep disturbance in women during the menopausal transition and post-menopause: data from the Norwegian prescription database and the HUNT study

**DOI:** 10.1186/s12905-020-00916-8

**Published:** 2020-03-30

**Authors:** Randi Andenæs, Milada Cvancarova Småstuen, Nina Misvær, Lis Ribu, Ingvild Vistad, Sølvi Helseth

**Affiliations:** 1 Faculty of Health Sciences. Department of Nursing and Health Promotion, Oslo Metropolitan University, Oslo, Norway; 2 Sørlandet Hospital, Kristiansand, Norway; 3 Department of Clinical Science, University of Bergen, Bergen, Norway

**Keywords:** Sleep initiation and maintenance disorders, Estrogen, Menopause, Postmenopause, Female

## Abstract

**Background:**

Impaired sleep is common in menopausal women. The aim was to examine associations between uses of systemic menopausal hormone therapy (MHT) and sleep disturbance in a large population sample.

**Methods:**

Female participants aged 45 to 75 years were selected from the Norwegian Health Study in Nord-Trøndelag (HUNT3, 2006–2008) (*N* = 13,060). Data were linked to the Norwegian Prescription Database, identifying use of prescribed MHT and use of sleep medication. Data were analyzed using multiple linear regression.

**Results:**

In total, 996 women used systemic MHT (7.6%), with the highest prevalence of 10.3% among women 55 to 64 years of age. Despite high reports of frequent nocturnal awakening (24.7%) and high reports of hot flashes, use of MHT was low in this large population based survey. Although MHT use was associated with more sleep disturbance in unadjusted analyses, the association was not significant after adjusting for relevant covariates. Using sleep medication, reporting poor health, tobacco and alcohol use, doing daily exercise, having higher levels of anxiety, and being less satisfied with life were factors showing the strongest associations with sleep disturbance.

**Conclusion:**

The lack of association between MHT and sleep disturbance suggests that other factors, such as self-perceived good health, a healthy lifestyle and anxiety/depression, are more relevant to sleep than MHT.

## Background

Good sleep is a key factor for the maintenance of quality of life and sleep disturbance has been recognized as an important public health concern [1]. Sleep quality changes throughout life, influenced by various factors such as biology, hormones, lifestyle, and age. Menopause, with declining estrogen levels that mark the end of the reproductive phase of a woman’s life, is considered a particularly vulnerable period of life with regard to sleep quality [2, 3]. Impaired sleep quality is also reported in postmenopausal women [4, 5], related to physiological changes during late adulthood and old age.

The prevalence of poor sleep in menopausal women shows some variation. In the Study of Women’s Health Across the Nation (SWAN), Kravitz and colleagues [2] followed a multiethnic sample of 12,603 women for 10 years prospectively, and difficulties falling asleep, staying asleep, or early morning awakening were reported over the course of seven annual assessments. Overall, menopause was associated with the onset of sleep disturbances, with sleep problems present in 46 to 48% of menopausal women versus 38% of pre-menopausal women [2]. In a literature review, Polo-Kantola (2011) reported that among women aged 50 to 64 years, 25% had sleep problems, and 15% of these had severe sleep disturbances that substantially affected their quality of life [6].

Possible explanations for the worsening of sleep disturbance that occurs during the menopausal transition and postmenopausal years include vasomotor symptoms (VMS), which probably relate to the natural decline in estrogen levels [7, 8]. Women with VMS typically suffer from perspiration or palpitations during the night and may experience frequent awakening with sweating [6]. These episodes are described as hot flashes or night sweats, which often, but not always, awaken the menopausal woman [9]. VMS tend to fluctuate and their severity varies greatly between individuals, with some reporting intense discomfort that to a greater or lesser degree reduces quality of life [10] and impairs health status [11]. Longitudinal research suggests that the time from onset to resolution of VMS may be more than 7 years [12]. VMS are one of the chief menopause-related problems for which women seek medical treatment [13].

Other complaints, such as mood disorders, especially depression and anxiety, which may be secondary to vasomotor symptoms or related to other causes, are common during menopause and typically cause sleep problems [14]. Prospective studies have observed an increased risk for the development of significant depressive symptoms in women with no history of depression entering perimenopause compared with age-matched women who remained premenopausal [15, 16]. Lifestyle factors such as smoking and alcohol also reduce sleep quality [17].

Menopausal estrogen deficiency can be treated with systemic hormone therapy, either as estrogen alone (primarily in women who have had their uterus removed) or combined estrogen-progestogen therapy. Evidence from a recent systematic review evaluated the effect of menopausal hormone therapy (MHT) on self-reported sleep outcomes when compared to placebo in postmenopausal women, suggest that MHT benefits sleep in women with VMS, while for those without such symptoms, the effect is uncertain [18]. However, among the 42 randomized clinical trials included in the review, of which seven included sleep quality as a primary outcome measure, the majority lacked a baseline screening for sleep disorders, and report of prior medication use to aid sleep was scarce. Further, behavioral factors (e.g. smoking, little physical activity) may also contribute to reduced sleep quality in women’s midlife years [19, 20].

Given that evidence of the association between use of MHT and sleep is inconclusive, there is a need for more insight from studies of unselected samples of women, representing a wider age range. There is a particular paucity of research among women older than 64 years. To address these gaps in the research literature, we examined the associations between sleep disturbance and the use of MHT among women aged 45 to 75 years of age. Other covariates, such as VMS, self-evaluation of health, satisfaction with life, anxiety and depression, use of sleep medication, and selected lifestyle factors (smoking, alcohol, frequency of exercise, and partnership) were also evaluated.

## Methods

### Study design and sample

**Fig. 1:**
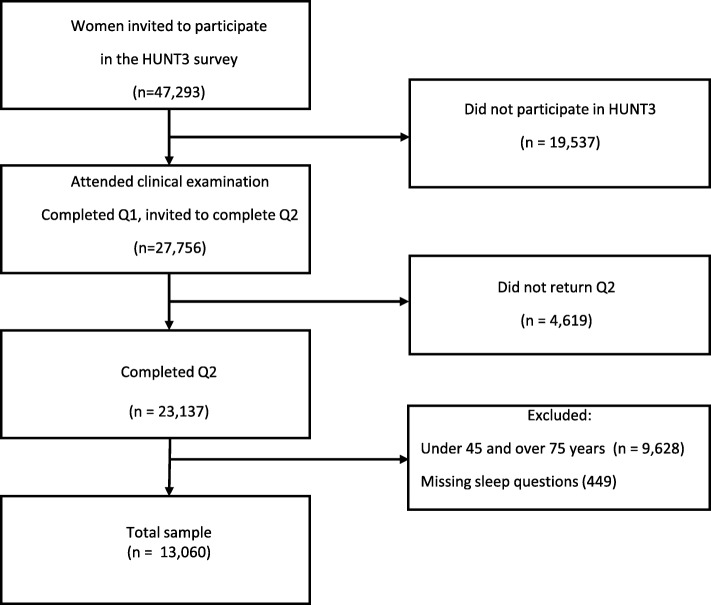
Flowchart of respondents

We received data from the Nord-Trøndelag Health Study (HUNT); which is a large health survey in Norway [21]. In the third survey, HUNT3, carried out in 2006–2008, all eligible inhabitants aged 20 years or older living in the county of Nord-Trøndelag were invited to participate, constituting 93,860 individuals; including 47,293 women. In local field stations, a general clinical examination took place, and those who chose to participate in HUNT 3 completed questionnaire 1 (Q1) with basic socio-demographic data (age, education, cohabitation) and common health conditions. At the field station, a second questionnaire (Q2) was distributed in HUNT 3, in which questions about sleep and issues related to the menopausal transition were included. Q2 was answered at home and returned by email. A total of 27,756 women (58.7%) attended the clinical examination and answered Q1, and of these, 23,137 answered Q2. In the present analyses we included 13,060 women between 45 and 75 year with complete sleep variables (Fig. 1). The HUNT survey is fairly representative of the general Norwegian population [22].

### Measures

#### Sleep disturbance

The Insomnia Index in the HUNT [23] was used to measure sleep problems. The following questionnaire items are included in the index: During the last 3 months how often have you; 1) had difficulties falling asleep at night, 2) woken up repeatedly during the night, and 3) awoken early and not been able to fall asleep again. Response options were never/rarely, sometimes, or several days a week. Sum scores were computed to constitute sum scores, ranging from 3 to 9, where higher scores indicate more sleep problems. Cronbach’s α in the current sample was 0.69.

#### Menopausal hormone therapy, sleep medications and VMS

In Norway, when a prescription from a clinical doctor is dispensed from a pharmacy, the drug information is sent electronically to the NorPD. Participants in the HUNT3 had consented to their individual study data being linked to official registers. By providing their individually unique national registration number, NorPD data for the 2007 calendar year were merged with the HUNT3 data by the Norwegian Institute of Public Health and the HUNT Research Centre. Prescribed medications followed the Anatomical Therapeutic and Chemical (ATC) classification system classes and codes [24]. MHT included estrogen alone (ATC codes G03C A03 and G03C X01), as well as the combination of estrogen and progesterone (G03F A01, G03F A12, and G03F B05). Systemic MHT included oral administration and transdermal patches, and while local vaginal applications are also reported, they were not included in the regression analysis. MHT is used as an umbrella term to describe both estrogen alone and the combination of estrogen and progesterone, and we categorized the sample into two groups: users (if systemic MHT was received from a pharmacy at least once) and non-users (if no systemic MHT was received).

Sleep medications included benzodiazepine-derivate anxiolytics (N05B A01, N05B A04, and N05B A09), benzodiazepine-derivate hypnotics (N05C D02) and Z-drugs (N05C F01, and N05C F02). Although benzodiazepines have a mix of different pharmacological actions, these medications are all indicated for sleep problems. In the regression analysis, anxiolytics and hypnotics were therefore aggregated into a single category of sleep medication. Users had received one or more prescription of sleep medication, and non-users had received none.

Measurement of physical VMS included hot flashes and night sweats. The question “Do/did you experience hot flashes during menopause” had the following response options: ‘during the day’, ‘during the night’, ‘both during the day and night’, or ‘never/no report’. The follow-up question “If you experienced hot flashes, how bothersome did you find them”, had the response options: ‘major’, ‘medium’, or ‘small’. Having bothersome night sweats during the last 3 months was reported as ‘never/rarely’, ‘sometimes’, or ‘several times a week’.

#### Covariates

Covariates selected from the HUNT3 database were based on literature review, and identification of factors associated with sleep quality. Information on sex, age, and cohabitation (living with a partner/others versus living alone) were included. The frequency of physical activity was defined as number of times the person exercised last week; never, less than once a week, once a week, 2–3 times a week, or daily. The questions were supported with examples of common types of exercise (e.g. strolling, skiing, swimming or more vigorous training (e.g., sports). Smoking habits were defined as non-smoker (never smoked or former smoker) or current smoker (smokes daily or now and then). Responders to alcohol questions were categorized into abstainers or not. Anxiety and depression were assessed with the Hospital Anxiety and Depression Scale (HADS) [25]. The scale has seven questions for anxiety and seven for depression, and the frequency of each symptom is rated from 0 (not at all) to 3 (very often), resulting in sub-scores between 0 and 21 for anxiety and depression. Higher scores indicate more anxiety/depression. Satisfaction with life was measured by a single item: “When you think about how you are feeling at the moment, are you mostly happy with life or are you mostly dissatisfied?” with a seven-point Likert style scale, ranging from ‘very satisfied’ to ‘very dissatisfied’. Self-assessment of health was measured with the single item ‘How is your health in general?’ with response options of bad, fair, good, and very good. Both single item questions have been widely used in other epidemiological studies [26, 27].

### Statistical analyses

Data are described with the means and standard deviation for continuous variables and with frequencies and percentages for categorical data. The associations between sleep quality disturbance and selected variables were analyzed using univariate and multiple linear regression models. Variables that were associated with sleep – based on the literature and our clinical knowledge - were included in the multiple regression model. The results are presented as regression coefficients (B) with 95% confidence intervals (CI). The assumptions for linear regression were fulfilled as the residuals were normally distributed. All analyses were considered exploratory, so no correction for multiple testing was performed and *p*-values < 0.05 were considered statistically significant. All statistical analyzes were carried out using SPSS, version 25 (IBM Corp, Armonk, NY).

### Ethical approval and consent to participate

HUNT3 was approved by the Regional Committee for Medical Research Ethic (REK), Mid-Norway. The participation is voluntary, and all participants signed written informed declaration and consent before inclusion in the study. All future research projects must gain approval from the ethical committee, and this study was approved by REK (2012/2264) and the Norwegian Social Science Data Service (NSD) (16/00284–4). The NSD gave their permission to linkage NorPD data from the Norwegian Institute of Public Health (PDB 1582) with data from the HUNT Research Centre. Participants in the HUNT3 had also provided an extended informed consent, which allowed linkage between the databases.

## Results

The demographic characteristics of the study participants are presented in Table 1. More than one third of the participants reported night sweats, and almost half had experienced hot flashes both during night and day. The mean score on the Insomnia Index was 5.6 (scoring 3–9).

**Table 1:** Sample characteristics (*N* = 13,060)

	N	%
Age, years (in categories)		
45–54	4313	35.8
55–64	4700	39.0
65–75	3026	25.2
Cohabitation		
Living alone	2594	19.9
Living with partner/others	10,466	80.1
Exercise		
Daily	2676	20.8
2–3 times a week	5679	44.0
Once a week	2521	19.6
Less than once a week	2015	15.6
Smoking status		
Non-smoker	9402	74.0
Current smoker	3302	26.0
Night sweats		
Never/seldom	8111	62.4
Sometimes/often	4881	37.6
Hot flashes^a^		
Did not notice	4652	35.6
During the day	1229	9.9
During the night	891	6.8
Both during day and night	6213	47.7
Sleep medication		
Non-users	10,536	80.7
Users	2524	19.3
Life Satisfaction		
Very satisfied	2510	19.5
Quite satisfied	4621	35.8
Satisfied	4143	32.1
Dissatisfied	1628	12.6
Self-perceived health		
Very good	1615	12.8
Good	7067	56.0
Bad/fair	3947	31.2
	**mean**	**SD**
Hospital Anxiety and Depression Scale (HADS)		
Anxiety (scoring 0–21)	4.3	3.5
Depression (scoring 0–21)	3.3	2.9
Insomnia Index (scoring 3–9)	5.6	1.6

Note: ^a^Missing 45.7% in age group 45–54 yrs.

The most frequent sleep problem was nocturnal awakening, reported by one out of four several times a week (Table 2). The majority perceived however their health as good, and were satisfied or quite satisfied with their life.

**Table 2:** Descriptives for sleep problems in the Insomnia Index

Item	N (%)		
How often in the last 3 months have you had difficulties with			
	Never/seldom	Sometimes	Several times a week
Falling asleep	4557 (34.7)	6325 (48.5)	2178 (16.8)
Nocturnal awakening	3066 (23.4)	6773 (51.9)	3221 (24.7)
Too early awakening	5125 (39.1)	6098 (46.7)	1837 (14.1)

As shown in Table 3, a total of 996 (7.6%) women were using systemic MHT, with oral tablets being the most frequent mode of administration (6.9%, *n* = 907), and a smaller proportion using transdermal administration (patches) (0.4%, *n* = 46). Use of a local estrogen application (vaginal tablets) was reported by 1.117 (8.6%) women, out of which 42 (0.3%) also used oral or transdermal MHT. As the purpose of this study was to assess possible association between sleep disturbance and use of systemic MHT, women who used local application were considered non-users of MHT for the purpose of analysis. Systemic MHT was used by 6.8% of women 45–54 years of age, by 10.3% of women 55–64 years, and by 4.6% of women 65–75 years. About 20% used sleep medication, with most frequent use in the eldest age group.

**Table 3:** Use of MHR and sleep medication by age group

	MHT tablets and/or patches		
	Non-users N (%)	Users N (%)	Total N
Age category			
45–54 years	4413 (93.2)	321 (6.8)	4734
55–64 years	4549 (89.7)	525 (10.3)	5074
65–75 years	3102 (95.4)	150 (4.6)	3252
All	12,063 (92.4)	996 (7.6)	13,060
	Sleep medication		
	Non-users N (%)	Users N (%)	Total N
Age category			
45–54 years	4446 (87.6)	558 (12.4)	4734
55–64 years	4024 (79.3)	1050 (20.7)	5074
65–75 years	2366 (72.8)	886 (27.2)	3252
All	10,536 (80.7)	2524 (19.3)	

About one fourth of women who reported that they experienced problems due to their hot flashes described their problems as major, 23.4% in the youngest age group (44–54), 54.0% of those aged 55–64 and 22.6% in the oldest age group (65–75). Among MHT users, 52.2% reported major VMS, 28.4% medium VMS, and 6.4% minor VMS symptoms.

### Sleep disturbance

There was a strong association between use of MHT and use of sleep medication (*p* < 0.001). Of those who used MHT about 24.3% also used sleep medication compared to 18.9% of those who did not use MHT. In the univariate regression analyses (Table 4), there was a positive correlation between use of MHT and the Insomnia Index. Women who used MHT had higher scores on the Insomnia Index, indicating more sleep problems. When adjusted for age, anxiety, depression and satisfaction with life, there was still a significant positive correlation with the Insomnia index, indicating more sleep problems with MHT use. When further adjusting for self-assessed health and use of sleep medication, the effect of MHT was no longer significant. Further, age was significantly associated with sleep disturbance. Those in age group 55–64 and 65–74 had higher levels on the Insomnia index compared to the youngest age group. Of lifestyle factors, daily exercise, being a smoker and using alcohol, were all associated with sleep problems in the adjusted analyses. Both higher levels of anxiety and depression, and lower levels of life satisfaction, were associated with sleep problems. The association between good perceived health and sleep disturbance remained negative, also in multivariate analyses, indicating that women who reported better health had less sleep problems.

**Table 4:** Multiple linear regression analysis with sleep disturbance as dependent variable

	Univariate			Multivariate		
Independent variables	B	95% CI	*p-*value	B	95% CI	*p-*value
MHT (ref. = non-user)	0.14	0.03–0.24	< 0.001	0.04	−0.06 – 0.14	0.41
Sleep medication (ref. = non-user)	1.31	1.24–1.38	< 0.001	0.84	0.77–0.91	< 0.001
Age (years) (ref. = 45–54)						
55–64	0.38	0.31–0.44	< 0.001	0.22	0.27–0.76	< 0.001
65–75	0.39	0.32–0.44	< 0.001	0.20	0.13–0.28	< 0.001
Living with partner/others (ref. = living alone)	− 0.19	− 0.26 – − 0.12	< 0.001	− 0.03	− 0.10 – 0.04	0.43
Exercise (ref. = <once a week)						
Once a week	−0.17	− 0.26 – - 0.07	0.001	0.01	−0.08 – 0.10	0.85
2–3 times a week	−0.20	−0.28 – - 0.11	< 0.001	0.03	−0.06 – 0.10	0.62
Daily	−0.12	−0.21 – - 0.02	0.01	0.17	0.02–0.20	0.02
Non-smoker (ref. = current smoker)	0.01	−0.06 – 0.07	0.85	−0.18	−0.24 – − 0.12	< 0.001
Alcohol (ref. = no use)	− 0.11	− 0.18 – − 0.05	0.001	0.12	0.06–0.19	< 0.001
Hospital Anxiety and Depression Scale (HADS)						
Anxiety	0.15	0.14–0.16	< 0.001	0.12	0.11–0.13	< 0.001
Depression	0.06	0.04–0.07	< 0.001	0.02	0.01–0.03	0.002
Satisfaction with life (higher levels indicate less life satisfaction)	0.43	0.41–0.46	< 0.001	0.07	0.04–0.10	< 0.001
Self-assessed health (higher levels indicate better health)	−0.73	− 0.77 – − 0.68	< 0.001	−0.35	− 0.39 – − 0.30	< 0.001

## Discussion

In this population study, use of MHT showed no association with sleep problems in adjusted analyses, while using sleep medication, reporting anxiety/depression, smoking and alcohol use, doing daily exercise and lower levels of life-satisfaction were associated with more sleep problems. Reporting good health indicated less sleep disturbance.

The relatively low rate of MHT use evident in the present data from 2007 (7.6%) may be influenced by the findings from the Women Health Initiative (WHI) trial [28] and the Heart and Estrogen/progestin Replacement Study (HERS) II [29], which raised concerns about the risks and benefits of MHT. These studies reported a significant increase in breast cancer for users of MHT, without the expected primary preventive effect on cardiovascular disease and other chronic conditions. The studies resulted in a more cautious prescribing practice among doctors [30]. In the years following the WHI and HERS II studies, use of MHT declined from 44% of US women using or having used MHT in 1988–1994 to 4.7% of women in 2010 [31]. Norwegian gynaecologists’ attitudes toward MHT also shifted from being quite liberal to prescribe MHT, to becoming rather reluctant, after the aforementioned studies were published [32, 33]. Clinicians applied more strict indications for MHT, prescribed lower doses, and shortened the treatment duration of MHT [30]. The uncertainty regarding the actual cost vs. benefit to the user probably left many women reluctant to take MHT, or desisted from this type of treatment [33]. In more recent years, however, opinions regarding use of MHT have shifted away from the scepticism that came with the first WHI publications, as a result of new research, including a recent study on the long-term effects of the WHI study [34]. The latest recommendations from the North American Menopause Society [35] support use of MHT for treating bothersome VMS, but not for disease prevention. Consequently, our interpretations of the results bear these two perspectives, both historic and current, in mind.

There are some possible explanations for the lack of association between use of MHT and sleep in the present study. Our findings may confirm previous indications that MHT does not influence sleep quality: The Wisconsin Sleep Cohort Study (2003) of 589 premenopausal, perimenopausal and postmenopausal women found that postmenopausal women had the best sleep and this was somewhat worsened in women who were taking MHT [36]. In that study, the authors did not find evidence that hot flashes caused sleep disturbances. Other researchers also claim that the relationship between VMS and sleep disturbance during menopause is not well defined, and sleep problems are not necessarily due to VMS [37]. On the other hand, several studies report that sleep problems related to vasomotor symptoms can be improved with MHT [38]. Most trials comparing MHT with placebo have shown improvement in perceived sleep quality and self-reported sleep problems in women with VMS at baseline [18]. Other studies, such as an RCT comparing a low-dose antidepressant and low-dose MHT with placebo in menopausal women with hot flashes, showed that both medications modestly reduced symptoms of sleep problems compared to placebo [39]. The strong association between use of prescribed sleep medication and sleep problems in the present study probably reflects that women seek other treatments for their sleep difficulties, such as hypnotic drugs. This is in line with an earlier research that identified drug use (hypnotics and hormone therapy) as risk factors for sleep disturbance. Such use is suggested being caused by women with most menopausal symptoms, who despite a proven effect of the medication, still have more symptoms than observed in the general population [7].

In the present study, the reports of VMS in MHT users were high, with more than half of the women having major symptoms. The survey question in the HUNT3 was posed as the following; “Do/did you perceive hot flashes during menopause?” Positive answers could indicate that the women had a history of VMS without medication, giving a justification for their present prescription. The ambiguity of referencing either the past *or* the present time in the question gives support to this interpretation. A more likely explanation is that many women remain untreated, or do not receive adequate medication despite climacteric symptoms.

Of interest is that use of systemic hormone therapy was highest in the age group 55 to 64 years (10.4%), and was still used by women in the 65–75 years age group. Some may find it difficult or unnecessary to quit, as most women experience a reappearance of symptoms after treatment cessation [40]. When hormone therapy is recommended for women younger than 60 for the treatment of VAS and bone loss, for women aged 60 or older the risks of heart disease, stroke, venous thromboembolism, stroke and dementia become greater [35]. The 2017 hormone therapy position statement from The North American Menopause Society [35] recommends that clinicians individualize their decision-making, determine the proper dosage, and actively include the women in the shared decision-making process. Menopausal symptoms that interrupt sleep may be more troublesome than daytime symptoms and this should be considered when targeting therapy [41].

Anxiety and depression were significantly associated with poor sleep quality. Previous studies have identified mood disturbances as a strong predictor of poor sleep [42], and the relationship between sleep disturbance and depressed mood is likely bidirectional [43]. Although it is postulated that periods with hormone fluctuations across the female reproductive lifecycle represent ‘a window of vulnerability’ for depression [44], the low anxiety and depression scores reported in the present study are in line with the research literature that most women do not experience depression during menopause or in their midlife years [45].

In the adjusted analyses, our data showed no association between moderate frequency of exercise and sleep, except that doing daily exercise was associated with more sleep disturbance. Experience has also shown that daily exercise not necessarily remove vasomotor symptoms or provide good sleep. Earlier research about the effect of exercise on vasomotor symptoms and sleep is also inconclusive, and in a Cochrane review investigating the effectiveness of exercise in the treatment of VAS, the interventions differs in the different studies, due to e.g., content, intensity, frequency, and length [46]. According to the same Cochrane review, only a single, small study suggested that MHT is more effective than exercise to moderate VAS symptoms [46]. More research is needed within this area.

### Strengths and weaknesses

A strength of the study is the population-based approach with the inclusion of 13,060 women with self-reported data on sleep. Further, the study included important socio-demographic information, lifestyle, self-perceived health, and psychological factors, allowing for thorough adjustment of potentially confounding variables. Another strength is the use of a complete national prescription database (NorPD), which is not influenced by recall-bias and non-response.

However, our study also has some limitations. First, we were unable to exclude women for which MHT is contra-indicated, such as those with breast cancer or thrombosis, because these medical conditions are not specified in the HUNT database. Even though we lack this information, the numbers would be quite low, and we consider the risk of bias by this factor to be small. Another limitation is that we cannot be certain that the medication was actually taken as prescribed, but we have ensured that the prescriptions were filled at the pharmacy. Low adherence to MHT arises mainly from concerns about possible adverse effects [47]. Moreover, we do not have data on prescribed daily doses. However, women who experience a good therapeutic effect would most likely take their prescribed medication. Lastly, we don’t actually know whether the women were pre-, peri-, or post-menopausal, and whether MHT might have different associations with sleep in these different groups. The age groups were a proxy for these stages, but exact determination was not feasible for this type of population-based study.

## Conclusion

Despite reports of frequent nocturnal awakening and high reports of hot flashes, use of MHT was low in this large population based survey. The lack of association between use of MHT and sleep disturbance suggests that either did the women receive inadequate MHT, or that other factors such as self-perceived health, lifestyle and anxiety or depression were more relevant to sleep than MHT.

## Data Availability

The data that support the findings of this study are available from the NTNU/HUNT but restrictions apply to the availability of these data, which were used under license for the current study, and so are not publicly available. However, the data are available from the authors upon reasonable request and with permission of the NTNU/HUNT.

## Abbreviations

CI: Confidence interval
HADS: The Hospital Anxiety and Depression scale
HUNT: Nord-Trøndelag Health Study
MHT: Menopausal Hormone Therapy, systemic
OR: Odds ratio
VMS: Vasomotor symptoms
